# Laparoscopic Fundoplication Using the Excluded Stomach as a Novel Management Option for Refractory Bile Reflux Following One Anastomosis Gastric Bypass (OAGB)

**DOI:** 10.1007/s11695-021-05804-0

**Published:** 2021-11-24

**Authors:** Senarath Bandara Werapitiya, Senarath Pradeep Ruwanpura, Tanya Rochelle Coulson

**Affiliations:** St John of God Hospital, Corner Robertson Drive & Bussell Highway Bunbury, PO Box 5007, Bunbury, WA 6231 Australia

**Keywords:** One anastomosis gastric bypass, Refractory bile reflux, Fundoplication

## Abstract

**Background:**

One anastomosis gastric bypass (OAGB) is now a mainstream bariatric procedure. Refractory gastroesophageal reflux is a significant complication following OAGB, and conversion to Roux-en-Y has long been the treatment of choice for this issue. Strengthening the lower esophageal sphincter by Nissen fundoplication (NF) has been reported as an effective anti-reflux surgery. Here we report the short-term outcomes of a modified NF procedure using the excluded stomach (excluded stomach fundoplication—ESF) to treat refractory bile reflux in post-OAGB patients.

**Methods:**

Thirteen post-OAGB patients underwent ESF for refractory bile reflux during the study, as detailed in the surgical technique. This paper reports the 12 patients whose follow-up data are available.

**Results:**

Following ESF, the GERD-HRQL heartburn score improved from 22.7 ± 3.9 to 1.8 ± 3.5 (*p* < 0.05). The mean aggregate GERD-HRQL score improved from 27.9 ± 5.3 to 5.7 ± 5.9 (*p* < 0.05). The GERD-HRQL global satisfaction score showed that 100% of patients were satisfied with the improvement of symptoms. The mean VISICK score improved from 3.8 ± 0.39 to 1.2 ± 0.39 (*p* < 0.05). One patient was returned to the operating theatre to have the wrap loosened due to dysphagia. Eleven patients did not require PPIs after surgery.

**Conclusions:**

ESF significantly improved the VISICK score and GERD-HRQL of post-OAGB patients with refractory bile reflux in the short term. The current study is being continued to increase the sample size and the follow-up period.

## Introduction

Since Rutledge reported his experience with the first 1,274 cases in 2001 [1], OAGB has become the world’s third most performed bariatric procedure [2]. Gastroesophageal biliary reflux (biliary GERD) is an uncommon yet significant complication following OAGB [3]. Adverse consequences of biliary GERD, including erosive esophagitis and Barrett’s esophagus, have been well documented [4, 5], and the carcinogenic potential of bile in the stomach or esophagus have been debated in the past without substantial evidence supporting increased risks [6]. The incidence rate of post-OAGB refractory biliary GERD is reported to be 0.6–10% [7]. With the number of OAGB procedures increasing, the management of this rare complication is becoming a relevant topic.

Conversion to Roux-en-Y (RNY) has long been the treatment of choice for managing bile reflux following OAGB [8]. Nevertheless, RNY only attempts to divert jejunal contents from the stomach pouch rather than preventing reflux into the esophagus. A proper anti-reflux procedure like Nissen fundoplication (NF) aims to strengthen the lower esophageal sphincter (LES), preventing stomach contents from entering the esophagus, thereby preventing reflux. Anti-reflux surgery has been shown to reverse bile reflux adequately [9]. Nissen fundoplication is the most commonly performed anti-reflux operation [10].

As an alternative to the stomach fundus as a wrap, ligamentum teres and synthetic prostheses such as Angelchik prosthesis and LINX Acid Reflux Management system have been used with some success [11–13]. As detailed in the surgical technique, we trialled 360° fundal wrap using the excluded stomach with excellent initial results and coined the name “excluded stomach fundoplication” (ESF) for this procedure. At our institution, we have introduced this procedure as the routine for managing refractory bile reflux following OAGB in suitable patients. This paper aims to report the short-term outcomes of this novel procedure as an alternative to RNY.

## Patients and Methods

At St John of God Health Care (SJOGH), Bunbury, Western Australia, we have performed 850 OAGB procedures from December 2014 to June 2021. Out of these, 31 patients presented with one or more symptoms such as upper abdominal pain, heartburn, nausea, regurgitation or vomiting of bitter greenish-yellow fluid, throat irritation, cough, and hoarseness of voice. They were provisionally diagnosed as having GERD-like symptoms. All of them were treated with 40 mg esomeprazole daily for a minimum period of three months before re-assessment. Furthermore, all the patients were referred to the dietitian for further dietary and behavioural management, which included a trial of not going to bed until 90 min after the last meal and not having chocolate, alcohol or caffeine when relevant. Symptoms resolved in 14 cases with these measures. The remaining 17 patients were categorised as having refractory bile reflux. Out of these four patients underwent RNY before we introduced ESF at our centre. Thirteen patients underwent ESF from August 2019 to June 2021 and were followed up prospectively. One ESF patient was lost to follow up. This paper reports the short-term outcomes of the remaining 12 patients.

As a part of the patient preparation, a screening gastroscopy was performed. The pathologies we looked for were the presence of hiatus hernia, esophagitis, gastritis, and the presence of bile in the stomach and esophagus. During pre-operative gastroscopy, we carefully examined for the presence of gastro-gastric fistulae and marginal ulcers, both of which may give similar symptoms as bile reflux.

All procedures were carried out according to the ethical standards of the institutional and national research committee and in line with the 1964 Helsinki declaration and its later amendments or comparable ethical standards. This report is the result of a long term study approved by the ethics committee of SJOGH (#1432).

Patient demographics were recorded. GERD health-related quality of life (GERD-HRQL), a validated disease-specific quality of life questionnaire [14], and VISICK score [15] were used for assessment of the severity of symptoms. Intraoperative and postoperative complications were recorded. Improvement or resolution of bile reflux symptoms after the surgical intervention was determined by clinical resolution of symptoms using the postoperative VISICK and GERD-HRQL. A postoperative VISICK score between II and I has been associated with patient satisfaction [16]. The best possible aggregate score for GERD-HRQL is zero indicating the total absence of symptoms, whereas the worst is 50.

### Statistical Analysis

Variables with normal distribution were expressed as mean ± SD. The significance of differences of GERD-HRQL and VISICK scores before and after surgery were evaluated with a paired t-test and the Wilcoxon signed ranks test. Data were analysed using IBM® Statistical Package for the Social Sciences® (SPSS®) version 24. A *p*-value < 0.05 was considered statistically significant.

### Surgical Technique

**Fig. 1:**
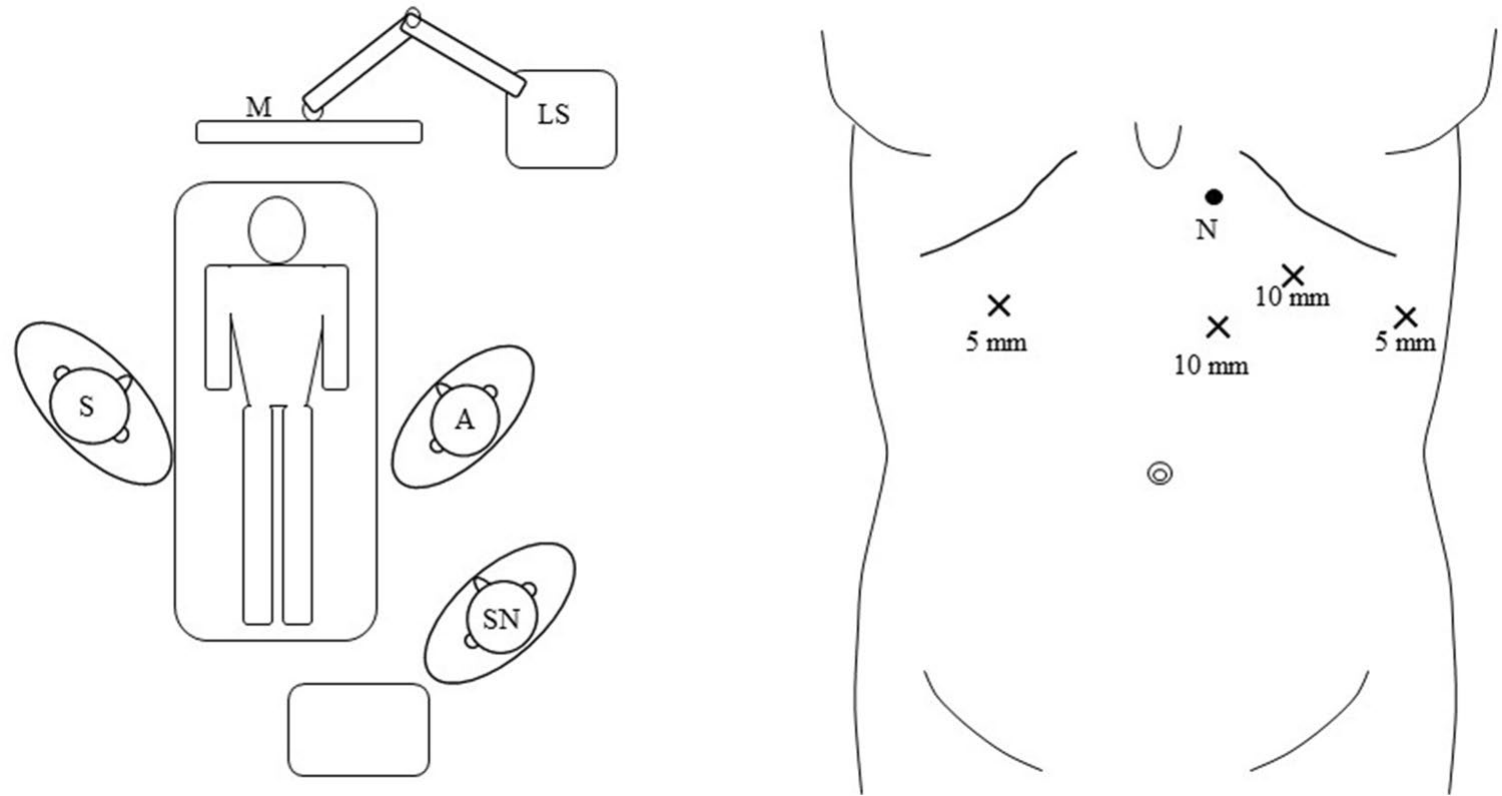
Patient position and placement of port sites for ESF. S, surgeon; A, assistant; SN, scrub nurse; LS, laparoscopic stack; M, monitor; N, port site for Nathanson liver retractor; X, other port sites and their sizes

All procedures were done under general anaesthesia with muscle relaxation. The patient was positioned supine with a 20° head-up tilt with the surgeon standing on the patient’s right. The peritoneal cavity was entered under direct vision using an optical trocar 4 cm below the left costal margin in the mid-clavicular line. Three other working ports and a Nathanson liver retractor were placed under laparoscopic vision (Fig. 1).

Careful assessment of anatomy was performed, paying attention to the configuration of gastro-enterostomy and the efferent jejunal limb. The efferent limb was followed for at least one meter to ensure no distal obstruction, promoting bile reflux. The stomach tube was then separated from the remnant stomach by dividing the adhesions using an ultrasonic energy device. The dissection was carried out to mobilise enough fundus for fundoplication by dividing up to 3 short gastric vessels.

**Fig. 2:**
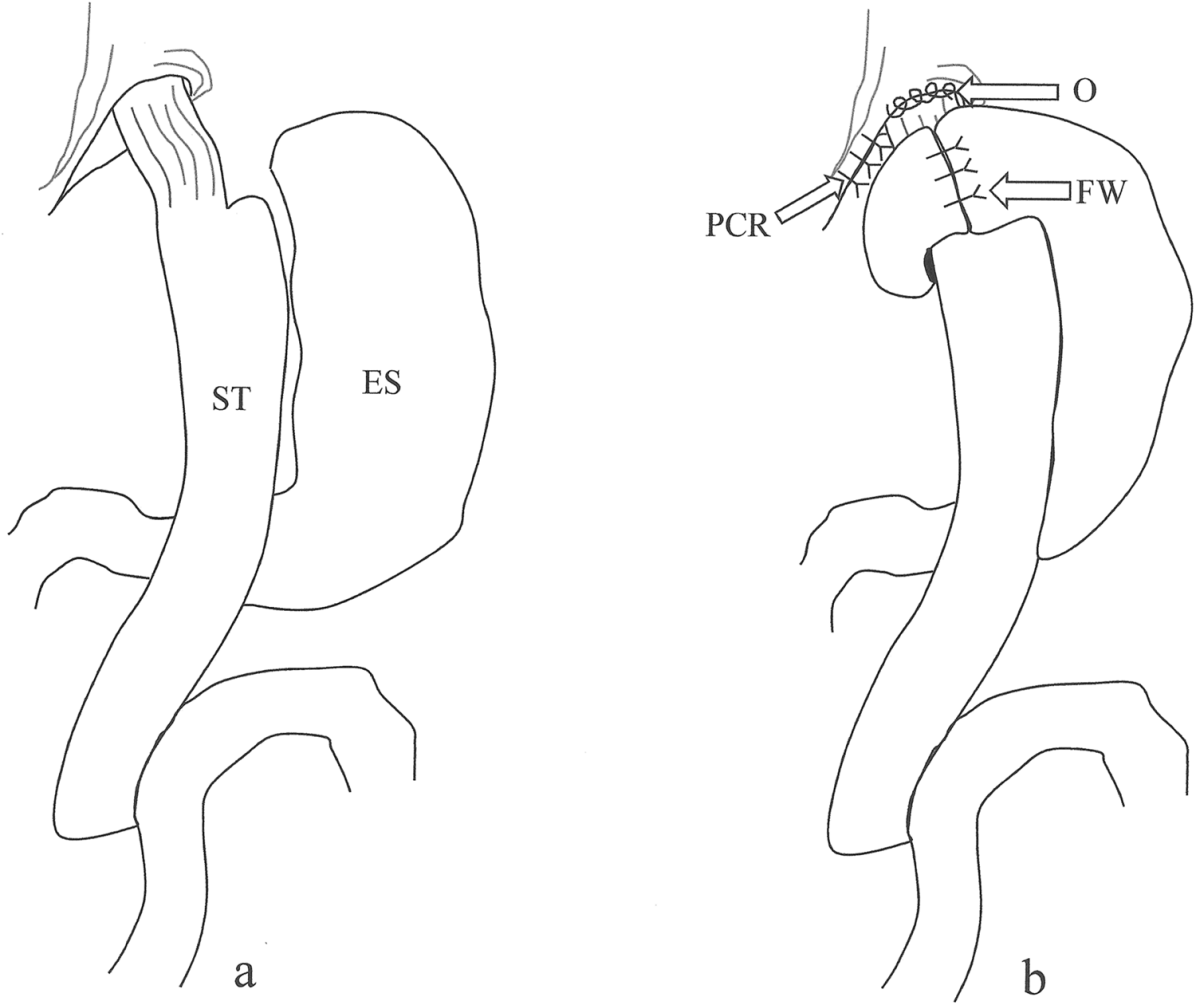
**a** Normal OAGB anatomy. ST, stomach tube; ES, excluded stomach. **b** Post-surgery. O, oesophagopexy; PCR, posterior crural repair; FW, 360° fundal wrap

A hiatal dissection was performed to demonstrate a hernia if present and to mobilise the distal esophagus. A posterior crural repair was fashioned snug over a 38F bougie using a non-absorbable suture. The phreno-esophageal ligament was recreated with bilateral esophagopexy using 2/0 V-Lock absorbable suture, maintaining a length of approximately 2 cm of the esophagus within the abdominal cavity. When a significant pouch was observed at the upper end of the stomach tube, this was excised in line over 38F bougie using a Covidien® tristapler pink cartridge. A 360° wrap was fashioned using the mobilised gastric fundus, loosely over the 38F bougie. This was done using three 0-Ethibond® gastro-gastric sutures with the upper and middle sutures attaching the wrap to the right crus and the esophagus, respectively (Fig. 2).

### Postoperative Management

Patients were maintained on intravenous (IV) fluids and an opioid patient-controlled-intravenous-analgesia (PCIA) for the first 24 h. Venous thromboembolism (VTE) prophylaxis was routinely provided by pneumatic intermittent calf compressors and 5000 international units of subcutaneous heparin twice daily. Essential oral medications for other conditions were maintained without interruption. A thin fluid diet was provided, and IV fluids were discontinued when oral intake was adequate.

## Results

The mean follow-up time in months was 11 ± 6 months. The baseline characteristics of the patients are summarised in Table 1.

**Table.1:** General characteristics of the study cohort

Gender male/female	2/10
Mean age ± SD	47.5 ± 13.2
Mean BMI (range) at ESF	29.35 (20.78–38.86)
Mean %WL (range) since OAGB	31.16 (9.47–47.92)
Mean follow-up period months (range)	11 (1–23)
Mean onset of reflux symptoms following OAGB months (range)	8.9 (0–42)
Mean duration of PPIs treatment before ESF months (range)	25.2 (3–61)

Before ESF, nine patients (75%) had severe reflux symptoms both night and day, whereas three patients (25%) complained that the symptoms were more severe at night. Nine patients had minimal improvement with proton pump inhibitors (PPIs) use but were left with significant residual symptoms affecting their quality of life. Out of 12, four experienced reflux predominantly within 3 h post-prandial. Eight patients (66.7%) had experienced symptoms thought to be due to aspiration of the regurgitate, and three required antibiotic treatment for chest infection.

Pre-operative gastroscopy showed that two out of 12 patients had erosive esophagitis, one patient LA grade A and the other LA grade B. One patient showed evidence of bile in the esophagus, whereas eight patients (66.7%) had bile in the stomach tube. Six patients (50%) showed endoscopic evidence of gastritis. The presence of a hiatus hernia was appreciated at gastroscopy in five patients (41.7%), and an additional four cases were observed during surgery. During ESF, the posterior crural repair was done in 10 patients. Furthermore, excision of the upper-end stomach pouch was performed in six patients.

The GERD-HRQL can be divided into a heartburn score, a dysphagia score, and general quality of life score. The mean pre-operative GERD-HRQL heartburn score improved from 22.7 ± 3.9 to 1.8 ± 3.5 (*p* < 0.05) post-operatively, while the mean dysphagia score reduced from 2 ± 3.4 to 1.1 ± 2.4 and was not statistically significant (*p* = 0.25). The mean aggregate GERD-HRQL score improved from 27.9 ± 5.3 to 5.7 ± 5.9 (*p* < 0.05) post-operatively. The GERD-HRQL global satisfaction score showed that 100% of patients were satisfied with the improvement of symptoms post-operatively. The mean VISICK score improved from 3.8 ± 0.39 to 1.2 ± 0.39 (*p* ˂ 0.05). These results are illustrated in Table 2.

**Table.2:** Comparison of GERD-HRQL and VISICK scores before and after ESF

Scores (mean ± SD)	Pre-ESF	Post-ESF	*p*-value
GERD-HRQL heartburn score	22.7 ± 3.9	1.8 ± 3.5	0.00001
GERD-HRQL dysphagia score	2 ± 3.4	1.1 ± 2.4	0.25
GERD-HRQL total score	27.9 ± 5.3	5.7 ± 5.9	0.00001
VISICK score	3.8 ± 0.39	1.2 ± 0.39	0.001

The only significant postoperative complication encountered was the patient who returned to operating theatre on day three post-surgery to have the wrap loosened due to dysphagia. Eleven patients (92%) did not require PPIs after surgery, whereas one patient was still using medication on demand at the time of follow-up.

## Discussion

Out of 850 OAGB patients, 17 (2%) developed refractory bile reflux. This observation is consistent with previously published data [7]. This paper reports the 12 patients who underwent ESF and whose follow-up data are available.

We have OAGB follow-up data for up to seven years. In the present study, all but one patient developed reflux symptoms within the first 12 months after OAGB. The one exceptional patient started reflux symptoms 42 months following OAGB. There is no published literature investigating the time of onset of bile reflux after OAGB. However, Zarshenas et al. reported that the incidence of reflux following OAGB in 45 patients was highest during the first year [17]. Authors believe that a similar observation in 850 patients substantiates the notion that the onset of bile reflux after OAGB is far more common within the first year.

We observed bile in the esophagus in one patient and the gastric pouch in eight patients. The presence of bile in the gastric pouch is expected after OAGB. Authors believe that bile in the esophagus during gastroscopy can be due to suction applied at the time and has limited diagnostic value. Dixon et al. have reported the histological changes associated with bile reflux gastritis in 1986 [18]. The Sydney scoring system has described a bile reflux index (BRI) to grade the resulting gastric mucosal changes [19]. Nevertheless, histologic findings including foveolar hyperplasia or intestinal metaplasia in the stomach and esophagitis are not specific to bile irritation. To date, no histological marker specific for bile reflux has been reported. Principal studies reporting bile reflux following OAGB have diagnosed bile reflux based on clinical symptoms [20], and we adopted a similar approach. Ten of our patients reported yellow/green bitter reflux indicating bile. Furthermore, lack of symptomatic control by PPIs makes acid reflux an unlikely cause, favouring a diagnosis of bile reflux in this cohort. Ten out of 12 patients showed no significant erosive esophagitis despite symptoms, making it unlikely they had predominant acid reflux.

Anti-reflux surgery aims to establish adequate LES pressure [21]. Nissen fundoplication has been reported as an effective treatment in the medium and long-term in most patients [22]. A recent study reported reasonable patient satisfaction and durable symptomatic relief up to two decades after laparoscopic fundoplication for GERD [23]. It has been reported that Nissen fundoplication improves VISICK [24, 25] and GERD-HRQL [26, 27] scores of patients with GERD. Stein et al. reported that NF could suppress bile reflux into the esophagus entirely [9]. Consistent with reports on standard NF results, our patients showed a significant improvement in GERD-HRQL and VISICK scores following ESF. Aggregate mean GERD-HRQL scores improved from 27.9 ± 5.3 to a score of 5.7 ± 5.9 while, mean VISICK score improved from 3.8 ± 0.39 to 1.2 ± 0.39. Out of GERD-HRQL components, the dysphagia score did not improve significantly. Due to the nature of fundoplication, improvement of dysphagia is not expected. In fact, Post fundoplication dysphagia has been reported as an adverse effect, with an incidence rate of up to 23% [28]. Only one of our patients developed de novo dysphagia post ESF.

During the ESF procedure, in addition to the 360° fundal wrap, we performed a crural repair in 10 patients. Recreation of the phreno-esophageal ligament was done to maintain a length of the esophagus in the abdomen. Furthermore, excision of the stomach pouch was performed to prevent liquid pooling in the most dependant part of the stomach in six patients. The authors believe that all these steps have the potential to contribute to the improvement of symptoms.

Since fundoplication achieves an actual strengthening of LES, there are many advantages of fundoplication over RNY in this setting. Fundoplication not only achieves symptomatic relief of reflux but also alleviates the undesirable long-term exposure of esophageal mucosa to potentially harmful stomach content, including acid, bile, and digestive enzymes. In comparison, fundoplication as a stand-alone procedure eliminates the need for a new staple/suture line avoiding the associated risk of leak/and bleeding. Unlike RNY, Fundoplication does not add to the length of the bypass, which may be undesirable for patients who have already achieved desirable weight loss. Fundoplication also avoids RNY related complications such as internal hernia [29] and afferent loop syndrome [30]. In addition, conversion to RNY makes reversal or revision more challenging. Preserving OAGB anatomy in ESF makes these options readily available. Furthermore, for patients with existing severe reflux or Barrett’s disease, ESF may be performed during the OAGB procedure as a preventive measure for reflux. The only possible disadvantage of ESF over RNY is that ESF will not prevent bile reflux into the stomach tube, leaving the potential to develop biliary gastritis symptoms.

Six patients in this cohort had OAGB as a revisional procedure following a previous gastric band. Although a previous band makes hiatal surgery more complicated, we maintained a very low complication rate. For fundoplication, the reported perioperative mortality rate ranges from 0.1 to 0.2% with an acute complication rate of up to 4% and the highest prolonged complications rate stands at 30% [31]. Our patients had zero mortality rate and no intraoperative complications. Only one patient (8.3%) had to be re-operated due to the tightness of the wrap. At the time of writing this paper, no prolonged complications had been reported, which makes us think this procedure is safe.

Our data demonstrated that ESF offered excellent control of bile reflux symptoms and improved health-related quality of life of post-OAGB patients in the short term. The authors propose ESF as a promising procedure in treating refractory bile reflux in post-OAGB patients. The current study is being continued to increase the sample size and the follow-up period.

The notable limitation of our study was the lack of specific diagnostic methods to differentiate between acid and bile reflux and to measure the LES function. Such data could have been correlated to symptoms and added more value to this report.

## Conclusion

Fundoplication using the excluded stomach significantly improved the VISICK score and GERD-HRQL of post-OAGB patients with refractory bile reflux in the short term. This procedure can be performed safely with an acceptable rate of complications.
